# Social and Self-Reflective Use of a Web-Based Personally Controlled Health Management System

**DOI:** 10.2196/jmir.2682

**Published:** 2013-09-23

**Authors:** Annie YS Lau, Adam G Dunn, Nathan Mortimer, Aideen Gallagher, Judith Proudfoot, Annie Andrews, Siaw-Teng Liaw, Jacinta Crimmins, Amaël Arguel, Enrico Coiera

**Affiliations:** ^1^Centre for Health InformaticsAustralian Institute of Health InnovationUniversity of New South WalesSydneyAustralia; ^2^UNSW MedicineUniversity of New South WalesSydneyAustralia; ^3^Black Dog Institute and School of PsychiatryUniversity of New South WalesSydneyAustralia; ^4^UNSW Counselling and Psychological ServicesUniversity of New South WalesSydneyAustralia; ^5^Centre for Primary Health Care and EquitySchool of Public Health and Community MedicineUniversity of New South WalesSydneyAustralia; ^6^University Health ServiceUniversity of New South WalesSydneyAustralia

**Keywords:** personal health record, social networks, Internet intervention, health service, help-seeking, emotional well-being, physical well-being, preventative health, eHealth, consumer, university

## Abstract

**Background:**

Personally controlled health management systems (PCHMSs) contain a bundle of features to help patients and consumers manage their health. However, it is unclear how consumers actually use a PCHMS in their everyday settings.

**Objective:**

To conduct an empirical analysis of how consumers used the social (forum and poll) and self-reflective (diary and personal health record [PHR]) features of a Web-based PCHMS designed to support their physical and emotional well-being.

**Methods:**

A single-group pre/post-test online prospective study was conducted to measure use of a Web-based PCHMS for physical and emotional well-being needs during a university academic semester. The PCHMS integrated an untethered PHR with social forums, polls, a diary, and online messaging links with a health service provider. Well-being journeys additionally provided information to encourage engagement with clinicians and health services. A total of 1985 students and staff aged 18 and above with access to the Internet were recruited online, of which 709 were eligible for analysis. Participants’ self-reported well-being, health status, health service utilization, and help-seeking behaviors were compared using chi-square, McNemar’s test, and Student’s *t* test. Social networks were constructed to examine the online forum communication patterns among consumers and clinicians.

**Results:**

The two PCHMS features that were used most frequently and considered most useful and engaging were the social features (ie, the poll and forum). More than 30% (213/709) of participants who sought well-being assistance during the study indicated that other people had influenced their decision to seek help (54.4%, 386/709 sought assistance for physical well-being; 31.7%, 225/709 for emotional well-being). Although the prevalence of using a self-reflective feature (diary or PHR) was not as high (diary: 8.6%, 61/709; PHR: 15.0%, 106/709), the proportion of participants who visited a health care professional during the study was more than 20% greater in the group that did use a self-reflective feature (diary: *P*=.03; PHR: *P*<.001).

**Conclusions:**

There was variation in the degree to which consumers used social and self-reflective PCHMS features but both were significantly associated with increased help-seeking behaviors and health service utilization. A PCHMS should combine both self-reflective as well as socially driven components to most effectively influence consumers’ help-seeking behaviors.

## Introduction

Personal health records (PHRs), tools that allow consumers to control and maintain their own health data and information, have been advocated as a next-generation technology that can significantly improve health behaviors and outcomes [1]. Well-designed PHRs can facilitate consumers’ self-reflection of their health by personalizing evidence-based guidelines, and providing judicious prompts, decision aids and tools to assist in maintaining their own health data and associated information. They currently form a crucial component in many large-scale national eHealth reform strategies worldwide.

Since the definitions of PHRs were clarified in 2006 [2], there has been a vast amount of survey, observational, cohort/panel, and anecdotal evidence regarding the benefits and satisfaction that PHRs provide for patients and consumers [3-11]. However, a recent scoping review on PHRs concluded that more research is still needed in order to evaluate the effectiveness of PHR implementations [12]. Specifically, there is currently a lack of understanding regarding (1) the uptake and sustainability of PHRs (eg, what motivates patients’ adoption and long-term use of a PHR), (2) how we can optimize the functionality and usability of these systems, and (3) whether PHRs play a beneficial role in supporting self-managed health care (ie, more quality evidence in the form of randomized controlled trials is needed) [12].

In parallel, the emergence of online social media means that the Internet is no longer being used only to search for information about diseases or treatments, but it is also being used to connect individuals previously unknown to each other (via asynchronous forums like PatientsLikeMe), and assist people to engage and keep in contact with their existing social networks (such as via Facebook). Social networks have been demonstrated to significantly inform our choices and affect our decisions in relation to health (eg, choice of hospital [13] and physical activity [14]), and social media interventions are now being developed (eg, via Facebook [15]), with preliminary findings on their feasibility and efficacy emerging in areas such as sexual health [16] and physical activity [17].

Considering these two major trends in consumer eHealth research (PHRs and online social networks), personally controlled health management systems (PCHMSs), which include a PHR, social networking features, self-management tools, and consumer resources, are rapidly being developed and deployed worldwide. While trials of PCHMSs are emerging in various health settings such as in vitro fertilization [18], hypertension [19], diabetes [20,21], influenza vaccination [22-24], medication accuracy [25], breast cancer management [26], physical and emotional well-being[27], and asthma [28], with some demonstrating acceptance and/or significant benefits among patients and consumers [18,20,21,23,26,27], there remains a lack of literature on how patients and consumers actually use these systems in their everyday settings and how the use of the PCHMS might affect their health behaviors and decisions.

Additionally, few studies have examined ways to incorporate social features in a PCHMS for the purpose of improving health behavior outcomes—where the social network *is* the treatment [29]. Earlier studies on systems such as PatientsLikeMe examined how patients in similar situations accessed each other’s personal health information [30], and how having access to others’ health outcomes and treatment decisions may have impacted their decisions in medication use and choice of doctors [31]. Our recent studies identified bundles of PCHMS features that were associated with increased help-seeking behaviors [27], and how consumers interacted with each other and health care professionals in a PCHMS [32]. To our knowledge, that was the only study identifying bundles of features in a PCHMS that were significantly associated with consumers’ help-seeking behaviors [27].

To date, it remains unclear how we can best integrate online social networking features and self-reflective tools (such as PHRs) into the design of PCHMSs in order to maximize consumers’ uptake, improve their health behaviors and outcomes, and facilitate their long-term use. In particular, few studies have examined the mutual relationship between self and the crowd in influencing one’s health behaviors. Utilizing a multimethod approach (statistical, content analysis, and social network analysis), the aims of this paper were as follows: (1) to measure how consumers used the most common *self-reflective* features in a PCHMS, (2) to measure how consumers interacted within the community created by the *social* features of the PCHMS, and (3) to provide recommendations on ways to engineer a *socially driven and self-reflective* PCHMS that would improve individual health behaviors.

## Methods

### Study Data

This paper utilized the data gathered during our 2011 study [27]. Previous analyses of this study have been conducted, which identified bundles of PCHMS features that were associated with increased help-seeking behaviors [27] and consumers’ patterns of usage for the social features of the PCHMS [32]. A full description of our study design is included in Multimedia Appendix 1.

### Trial Design and Participants

A single-group pre/post-test online prospective study was conducted over a university academic semester (July to November 2011) to examine how participants used a PCHMS to manage their physical and emotional well-being. Participants were included if they were aged 18 or over and had at least monthly access to the Internet and email. Full details of the study protocol and the instruments used to measure participants’ well-being and help-seeking behaviors (ie, COOP/WONCA charts [33], well-being self-ratings and lifestyle intention, health advice-seeking and health advice-providing networks, and help-seeking behaviors and health service utilization) are described in Multimedia Appendix 1.

### Healthy.me

The PCHMS (called Healthy.me) was iteratively developed between 2009 and 2013 at the University of New South Wales and was tested in settings such as in vitro fertilization, influenza vaccination, and breast cancer management [18,23,26]. The first version contained features such as journeys (which provide users with evidence-based, health-related information to promote engagement with clinicians and health services in an actionable way), a PHR, and online appointment booking with the university primary care service.

The version of Healthy.me (version 2.0) that was used in this study added online appointment booking with the university counseling services, a diary (private access by default but participants could set it to public for other PCHMS users to view), forums (moderated by a general practitioner [GP] and counselor), and polls. Full details of each PCHMS feature are described in Multimedia Appendix 1.

### Data Analysis

#### Analysis Methods

We utilized multiple methods (statistical, content analysis, and social network analysis) to quantify how participants used each of these social (forum, poll) and self-reflective (diary, personal health record) features in support of their physical and emotional well-being. Polls are considered social features of the PCHMS because they reveal behavioral norms within the group and they offer participants a quick and anonymous way to evaluate their behavior against the “norms” of the group. These features were selected for further analysis because they were found to be significantly associated with consumers’ help-seeking behaviors [27] or because consumers used them frequently and found them the most engaging [32].

Statistical analyses were used to provide a comparison of participants’ self-reported well-being, health status, health service utilization, and help-seeking behaviors based on their usage patterns of each PCHMS feature. Content analysis was employed to provide an overview of the topics and issues contributed/discussed by participants in online forums and diaries. Social network diagrams were constructed to examine the online forum communication patterns between consumers and clinicians. Social network analysis and visualization provide the tools to delve deeper into the social network, identifying the most active members of the community (via network centrality) and exploring the intergroup relationships that exist between consumers and clinicians (via reciprocity).

#### Statistical Data Analysis

For each social and self-reflective PCHMS feature, help-seeking behaviors and health service utilization during the study were compared using chi-square analysis between users and nonusers. Self-reported well-being and health status were compared pre- and post-study using the McNemar’s test and Student’s *t* test for paired samples. Usage of each PCHMS feature was summarized using descriptive statistics. Statistical analysis was performed using IBM SPSS Statistics 20. Tests performed were two-tailed and assumed a cut-off of *P*<.05 for statistical significance.

#### Diary Entry Coding

Diary entries were coded to analyze for participants’ topics of reflection, issues of concerns, and their past/present/future intended actions, where each entry could be coded multiple times within each code category. The coding process was informed by literature on young adults’ physical and emotional well-being concerns [34,35] and how people used blogging or online systems for health and self-reflection [35].

Pre-codes were created by consensus prior to data coding based on topics, issues, and actions derived from a random selection of 20% of diary entries. While reviewing each diary entry, open codes were created to record topics, issues, and actions that were not anticipated in the pre-coding scheme. The coding process was conducted by one author (AG) and another author (AL) independently coded 10% of diary entries. Interrater reliability on entries coded by both authors was considered good according to Cohen’s kappa statistic (κ=0.71).

Code categories for topics discussed include (1) physical only (eg, stomach pain, muscle cramp), (2) emotional only (eg, stress), (3) physical and emotional (eg, food, exercise, sleep), or (4) nonhealth (eg, finance, work/study, social, relationship). For participants’ use of the diary, code categories include (1) activity recording (eg, what they did on the day), (2) self-reflection (eg, reflecting on concerns, observations and actions), (3) goal setting (eg, recording plans and/or intentions to act), and (4) progress recording (eg, food diary, exercise diary). Participants’ actions recorded in the diary are coded according to (1) past action, (2) present action, and their (3) future intended action.

#### Forum Entry Coding

Three forums were available to participants: two were dedicated to men’s and women’s health issues, and the third forum was for discussion of general health issues related to lifestyle (Stay Healthy). Participants could seek answers from fellow participants, the GP, or the counselor on all three forums.

Posts on the forums were coded to analyze for topic of discussion and participants’ response type, informed by literature on interaction patterns found in online social network and question-and-answering websites [36,37]. The coding scheme was pre-determined, where a random selection of 20% of forum posts were used to develop the coding scheme iteratively until consensus was reached on coding rules and the definition of each category before coding commenced. The coding process was conducted by one author (NM) and another author (AL) independently coded 10% of forum posts. Interrater reliability on entries coded by both authors was considered good according to Cohen’s kappa statistic (κ=0.75).

Each forum post could be coded with more than one topic and participants’ response type. Code categories for discussion topics included (1) medical (ie, seeking for advice on a medical issue), (2) lifestyle (eg, dietary, exercise), (3) emotional well-being (eg, distress, stress), (4) women’s health (ie, topics specific to women issues, such as Pap smear), and (5) miscellaneous (ie, topics that do not fit into any of the above-mentioned areas). For participants’ response types, code categories included (1) asking a new/follow-up question, (2) providing advice/support/information, (3) sharing experience, and (4) expressing thanks.

#### Social Network Analysis

Social network analysis methods were used to classify the types of group-level behavior (patterns of communication within the forum community) and to consider the effects of group behavior on individual behavior, including the utilization of the PCHMS, and the decisions and behaviors of the participants. The metrics chosen for the analysis are typical indicators of forum behaviors—the relative importance of individuals within the network and the shift from one-on-one interactions to group discussions. We then discussed how the ongoing interactions in the social features of the PCHMS might improve utilization.

Degree centrality (an indicator of the relative importance of individuals in a social network) is calculated for each node in the network by counting the total number of connections to other nodes. Reciprocity (which indicates how common “conversations” are in a forum) was calculated by the proportion of connections that were returned. A reciprocal connection is one in which two people have responded to each other at least once each. To estimate the relative significance of the reciprocity in the networks, we tested the observed values against a random baseline using a method described elsewhere [38]. Social networks of online forum communication patterns among consumers and clinicians, namely a GP and a counselor, were analyzed using MATLAB 7.11.1 and illustrated using Cytoscape 2.8.2 [39].

## Results

### Participants

A total of 1985 participants met inclusion criteria and were recruited into the study. All completed the pre-study questionnaire. Of those, 709 (35.72%) completed the post-study questionnaire. Analyses were conducted on the 709 eligible participants who completed both the pre-study and post-study questionnaires. Of these, 80.7% (572/709) participants logged into the PCHMS at least once; where 93% (40/43) of those who posted on the forum, 60.0% (195/325) of those who answered a poll question, 90% (26/29) who wrote a diary entry, and 70.8% (75/106) of those who entered a PHR entry, logged into the PCHMS more than once.

Baseline and demographic characteristics of eligible participants are presented elsewhere [27]. During the study, 54.4% (386/709) of participants sought formal or informal help (for themselves or others) on physical well-being matters and 31.7% (225/709) for emotional well-being concerns [27]. Furthermore, 36.5% (141/386) of participants who sought help for a physical well-being matter and 32.9% (74/225) for an emotional well-being concern indicated their decision to seek help was influenced by other people. Among those who visited the university counseling service during the study, 54% (22/41) were first-time visitors.

For the 52 participants who self-rated as “extremely” bothered by their emotional problems at pre-study (as measured by the COOP/WONCA charts [33], which have been demonstrated to be a valid and feasible one-time screening assessment for mental disorders in primary care [33]), 44% (23/52) visited a health care professional for their emotional well-being during the study. On a scale from 1 to 5 (where higher scores indicate a poorer functional status), there was a significant improvement at post-study in these participants’ self-rated ability to conduct usual activities or tasks, both at work/study, or inside and outside the home (pre-study: mean 3.3 [SD 1.0]; post-study: mean 2.9 [SD 1.1]; *t*_51_=2.3; *P*=.028). At post-study, these participants also expressed being less bothered by emotional problems such as feeling stressed, anxious, depressed, irritable, or downhearted and sad compared to pre-study (pre-study: mean 5.0 [SD 0]; post-study: mean 2.6 [SD 1.1]; *t*_51_=9.5; *P*<.001).

### Social Features

Among the 709 participants eligible for analysis, the three features most participants accessed in the PCHMS were journey (84%, 95% CI 81-87), poll (46%, 95% CI 42-50), and forum (16%, 95% CI 13-19). Further, the poll and the forum (ie, the social features of the PCHMS) were the two features rated most frequently by participants as “useful” (poll: 32%, 95% CI 29-36; forum: 30%, 95% CI 27-34) and “fun or engaging” (poll: 35%, 95% CI 32-39; forum: 16%, 95% CI 13-19). Chi-square analyses showed that users of a PCHMS social feature (ie, poll or the forum) proportionally outnumbered nonusers in the following observed behaviors (see Table 1).

#### Forum

The most frequently posted topic category was “medical” for both the men’s health (64%, 14/22) and women’s health (61%, 20/33) forums. For the “Stay Healthy” forum, the most frequently posted topic category was “lifestyle” (52%, 15/29). Across all three forums, the most frequent interaction type was “providing advice/support/information”—men’s health: 72% (34/47); women’s health: 73% (40/55); and Stay Healthy: 46.6% (103/221). Chi-square analyses showed that forum posters proportionally outnumbered nonposters in the following observed behaviors (see Table 2).

**Figure 1:**
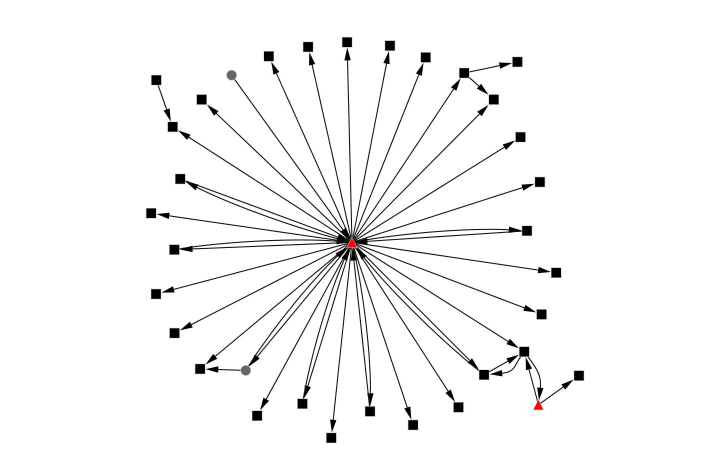
Networks for the Women’s Health forum. Black squares are female, grey circles are male, the Healthy.me GP and/or counselor is represented in the red triangle.

**Figure 2:**
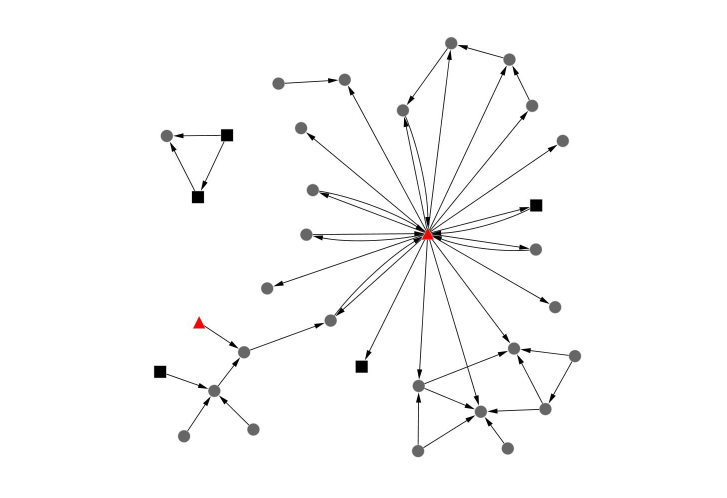
Networks for the Men’s Health forum. Grey circles are male, black squares are female, the Healthy.me GP and/or counselor is represented in the red triangle.

Table 3 outlines the social networking characteristics for the three forums. The women’s health forum most closely represented a star-shaped pattern (also known as the hub-and-spoke typology), centered on the GP (80%, 37/46 of the connections involved the GP) (Figure 1). The GP’s position reflects a question-and-answer structure (featuring high levels of reciprocity), suggesting that the level of engagement was one-on-one conversations rather than community-wide discussions. The men’s health forum was also centered on the GP but the level of engagement with other members of the forum was higher (48%, 22/46 of connections did not involve the GP) (Figure 2). This network also featured high reciprocity (39%, 18/46 of connections were returned), and all reciprocal connections involved the GP.

**Figure 3:**
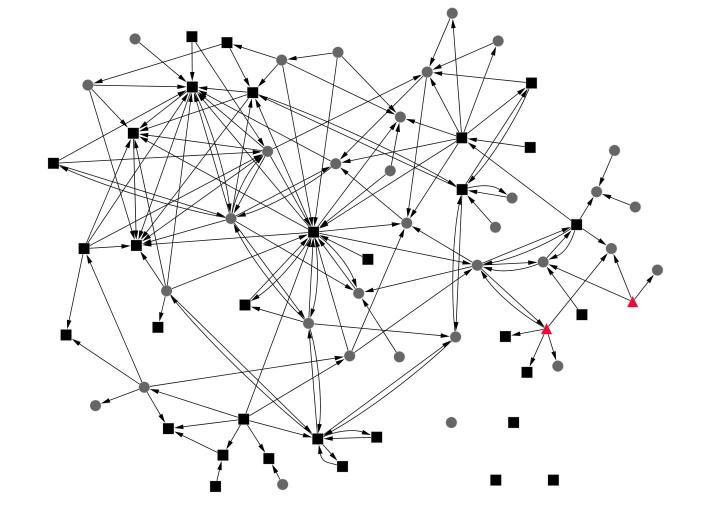
Networks for the Stay Healthy forum. Black squares are female, grey circles are male, the Healthy.me GP and/or counselor is represented in the red triangle.

Although there were some individuals with higher numbers of incoming connections, the Stay Healthy forum least resembled the star-shaped pattern and also featured high levels of reciprocity (28.8%, 42/146 of connections were returned) (Figure 3). However, clinicians in this forum did not play a central role (only 4.1%, 6/146 of connections involved the GP). The degree of centrality and the reciprocity in the Stay Healthy forum indicated a more conversational structure among participants compared to the men’s or women’s health forums.

#### Poll

Among participants who reported using the poll, 70.2% (174/248) reported that they enjoyed learning how their health compared with others, 41.1% (102/248) were surprised by others’ answers about their health, and 33.1% (82/248) reported that the poll changed their perception of how healthy they were or how healthy others were compared to themselves (ie, perceived themselves being healthier than others).

In addition, 13% (32/248) reported that using the poll changed some of their health actions and decisions. The results of McNemar’s test conducted on poll data shows that there was a significant increase in the number of participants reporting their perceived health as being better than others after using the poll compared to before usage (χ^2^_3_=41.57, *P*<.001). Chi-square analyses showed that poll users proportionally outnumbered nonusers in the following observed behaviors (see Table 4).

**Table 1:** Users of PCHMS social features (forum or poll) versus nonusers.

Observed behavior	Used social feature^a^, % (n) (n=332)	Did not use social feature, % (n) (n=376)	Difference, %	χ ^2^	*df*	*P*
Visited a health care professional	62.3 (207)	50.0 (188)	+12.3	10.9	1	.001
Sought formal/informal help for physical well-being concern	59.3 (197)	50.0 (188)	+9.3	6.33	2	.04
Self-rated being physically fit at post-study	91.9 (305)	84.6 (318)	+7.3	8.88	1	.003
Reported a higher intention to practice a healthy lifestyle at post-study	58.4 (194)	50.8 (191)	+7.6	4.14	1	.05
Had at least one person in their advice-seeking network at post-study	89.5 (297)	83.8 (315)	+5.7	4.86	1	.03

^a^Posted on forum or answered a poll question.

**Table 2:** Table 2. Users of online forum versus nonusers.

Observed behavior	Posted on forum, % (n)	Did not post on forum, % (n)	Difference, %	χ ^2^	*df*	*P*
Reported a higher intention to practice a healthy lifestyle at post-study	79.1 (34)^a^	52.8 (351)^b^	+26.3	11.25	1	.001
Visited a healthcare professional	63.4 (121)^c^	53.0 (274)^d^	+10.4	6.06	1	.01

^a^Out of 43 participants who posted on the forum.

^b^Out of 665 participants who did not post on the forum.

^c^Out of 191 participants who accessed the forum.

^d^Out of 517 participants who did not access the forum.

**Table 3:** Table 3. Social network characterization for the three forums.

	Women’s Health Forum	Men’s Health Forum	Stay Healthy Forum
Size, n	35	33	67
Density, %	3.8	4.2	3.2
Degree centrality of Healthy.me GP (% of connections)	37/46 (80%)	24/46 (52%)	6/146 (4.1%)
Reciprocity (% of connections)	18/46 (39%)	12/46 (26%)	42/146 (28.8%)
Reciprocity percentile (vs random baseline)	1.00	1.00	1.00

**Table 4:** Poll users versus nonusers.

Observed behavior	Answered poll question, % (n) (n=248)	Did not answer poll question, % (n) (n=460)	Difference, %	χ ^2^	*df*	*P*
Sought formal or informal assistance on an emotional health issue	37.9 (94)	28.3 (130)	+9.6	9.1	2	.01
Visited a health care professional	60.9 (151)	53.0 (244)	+7.9	4.1	1	.05

### Self-Reflective Features

#### Personal Health Record (PHR)

Approximately 15% of participants (106/709) added at least one entry into their PHR (ie, either an entry about their health schedule, medication, health care team member, pathology test result, imaging test results, or procedure). Altogether, 282 entries were added by participants during the study, with an average of 2.7 entries and a range of 1 to 12 entries per participant. Chi-square analyses showed that PHR users proportionally outnumbered nonusers in the following observed behaviors (see Table 5).

Examples of medication entries, health team members, and medical test results entered by participants are presented in Multimedia Appendix 2, Tables 1-5. Medication entries include prescription medications (eg, Zoloft), off-the-shelf products (eg, Vitamin C), and complementary medications (eg, Jasmine). Team members comprise GPs, allied health professionals (eg, chiropractor, osteopath, physiotherapist), specialists (eg, dentist, gastroenterologist, cardiologist, sports medicine physician), and nonclinical members (eg, boyfriend). Tests entered included blood tests (eg, full blood count, thyroid, ferritin/iron), urine tests (eg, dipstick for protein), screening tests (eg, pap smear, serum hepatitis B), and imaging tests (eg, mammogram, MRI, ultrasound, dental x-ray, lung x-ray, and bone-related x-ray). Both invasive and non-invasive procedures (such as mole removal, gall bladder removal, fractured ankle, vision test, LETZ, cryotherapy, right knee arthroscopy, mammogram, and colonoscopy) were also entered by participants.

#### Schedule and Online Appointment Booking Service

A total of 8.0% (57/709) of participants used the online appointment booking feature on the PCHMS. Chi-square analyses showed that users of online-appointment booking service outnumbered nonusers for the following behaviors (see Table 6).

#### Diary

Almost 9% (8.6%, 61/709) of participants completed at least one diary entry in the PCHMS. A total of 140 diary entries were written by 61 participants, covering 272 topics, 271 uses, and 272 actions. These participants on average wrote 2.3 entries (SD 4.9; mode 1.0), ranging from 1 to 38 entries per person. In a diary entry, participants expressed on average 2.0 topics (SD 1.1; mode 1.0; range of 1-6 topics), 2.0 issues (SD 1.0; mode 1.0; range of 1-5), and 2.0 actions (SD 1.1; mode 1.0; range of 1-5). No significant differences in the use of the diary (ie, topics/issues discussed, length of entries, number of entries per person) were detected between participants who sought help and those who did not seek help (*P*>.05), nor between those who reported an emotional/physical well-being concern and those who did not during the study (*P*>.05). Chi-square analyses showed that diary users proportionally outnumbered nonusers for the following behaviors (see Table 7).

Participants used the diary for both medical and nonmedical purposes, where the majority of entries were not medically related (Table 8). Most participants described their health concerns in terms of the encounters, changes, feelings, or symptoms they experienced in their everyday life (eg, food intake, feeling tired, light-headed, trouble going to sleep, crying) (Table 9). It was also uncommon for participants to use medical terminology to describe their experiences.

The diary was also used by participants to record past, present, and future-intended actions. Past actions include recalling past experiences (eg, remembering prior experience of ovarian cyst) and recording past activities (eg, saw a GP yesterday). Present actions include thought recording (such as concerns about bodily aches), reflection on relationships with family/friends, and discussion of current mood and state (eg, feeling down/sleepy). Future actions relate to activities that need to be completed (eg, reminder to book a medical appointment) or setting goals related to food intake and exercise routine.

**Table 5:** Personal health record (PHR) users versus nonusers.

Observed behavior	Entered PHR entry, % (n) (n=106)	Did not enter PHR entry, % (n) (n=602)	Difference, %	χ ^2^	*df*	*P*
Visited a health care professional	78.3 (83)	51.8 (312)	+26.5	25.61	1	<.001
Visited the university health service	28.3 (30)	16.3 (98)	+12	8.80	1	.003
Encountered someone who experienced a physical well-being concern	70.8 (75)	53.8 (324)	+17	10.51	1	.001
Sought formal/informal help for physical well-being concern	67.9 (72)	52.0 (313)	+15.9	9.22	1	.02
Sought formal/informal help for an emotional well-being concern	42.5 (45)	29.7 (179)	+12.8	6.74	1	.009

**Table 6:** Online appointment users versus nonusers.

Observed behavior	Booked appointment online, % (n) (n=54)	Did not book appointment online, % (n) (n=654)	Difference, %	χ ^2^	*df*	*P*
Visited the university counseling service	18.5 (10)	4.6 (30)	+13.9	18.16	1	<.001
Sought formal/informal help for an emotional well-being concern	44.4 (24)	30.6 (200)	+13.8	4.34	1	.04

**Table 7:** Diary users versus nonusers.

Observed behavior	Wrote diary, % (n) (n=29)	Did not write diary, % (n) (n=679)	Difference, %	χ ^2^	*df*	*P*
Visited a health care professional	75.9 (22)	54.9 (373)	+21.0	4.94	1	.03
Visited the university counseling service	20.7 (6)	5.0 (34)	+15.7	12.83	1	<.001
Encountered someone who experienced a physical well-being concern	82.8 (24)	55.2 (375)	+27.6	8.57	1	.003
Reported a higher intention to practice a healthy lifestyle at post-study	41.4 (12)	23.3 (158)	+18.1	5.00	1	.025

**Table 8:** Issues recorded by participants in their diary entries (n=140 entries).

Concerns / Intentions	Goals	Activities	Thoughts
Food	Study	Sleep diary	Feeling guilty about procrastination
Exercise	Fitness / diet plans	Food diary	Feeling worried about university progress
Physical and emotional well-being	Smoking cessation	Exercise routine	Brainstorming on food regimen
University demands		Symptom progress	Commenting on relationships
Relationship problems		Weight progress	Reflecting on daily experiences and new encounters
Finance		Daily activities	
Fatigue		University work	
Healthy eating/lifestyle		Actions taken towards physical and emotional well-being	
Seeking help for physical well-being concerns		Dietary intake	
Study plans			
Exercise/weight management			
Making plans for family/friends			

**Table 9:** Topics recorded by participants in their diary entries (n=140 entries).

Physical Health	Emotional Health	Physical and Emotional Health	Nonhealth
Headache/light-headedness	Post-traumatic stress disorder	Exercise	University
Nausea	Depression	Food	Work/life balance
Constipation	Mood swing	Sleep	Relationships (family, friends, romance)
Stomach pain/upsets	Anorexia nervosa	Diet	Daily activities
UTI/chlamydia screening	Anger	Weight	Social life
Injuries	Paranoia	Smoking	Finance
Cold/flu/cough	Panic	Binge eating	Hygiene
Ovarian cyst	Social anxiety	Fatigue/feelings of motivation	Death
Vaccines	Fatigue		Comments on Healthy.me
Sunburn	Sadness/crying		
Faint	Worry		
Back issues	Stress		
Vision	Feeling trapped		
Dental	Feeling lack of self-worth		
Skin rash			
Body aches			
Menstruation			

## Discussion

### Principal Findings

#### Social Versus Self-Reflective Features

This study compared how consumers used social and self-reflective features of a PCHMS. Our findings suggest that, although there is substantial variation in the use of social and self-reflective features, both are significantly associated with positive consumer health behaviors and outcomes.

#### Use of Social Features

In this study, social features (the poll and the forums) were two of the most frequently used features among consumers and were reported to be the most useful and most engaging. This study also contributes to our understanding of communication patterns in an online community and shows that these patterns can differ depending on the purpose of the social space (eg, medical advice seeking vs personal experience sharing) and the types of people participating in the space (eg, consumer vs clinician).

Findings on network centrality and reciprocity suggest that when it comes to consumers seeking answers to medical questions, the forum follows a star-shaped pattern with the GP in the middle. An interpretation of this pattern is that when a person with perceived “authority” (eg, a GP) contributes to a medically oriented discussion, other participants may see this as a definitive answer or may be less likely to contribute if the interaction is perceived as a personal patient-doctor interaction. However, when it comes to sharing lifestyle experiences, the forum did not follow a star-shaped network topology. Rather, consumers freely communicated with each other and the GP no longer played a central role of mediating the conversation in the forum.

#### Use of Self-Reflective Features

Although only 15.0% (106/709) of participants entered an entry into their PHR, this is the single feature that was significantly associated with consumers’ physical well-being help-seeking behaviors [27]. The use of the PHR, which encouraged participants to keep track of their personal health details (such as medication, test results, scheduled appointments, or health care team members), was significantly associated with more visits to a health care professional and help-seeking for physical well-being matters [27]. This may be related to increasing one’s self-efficacy by being aware of past and upcoming tasks and results [40].

Although only 8.6% (61/709) of participants used the diary, it was also one of the features identified as being significantly associated with emotional well-being help-seeking behaviors and visits to the university counseling service [27]. A diary, which encouraged self-reflection (in accordance with the principle of self-monitoring), is one of the most common behavioral change techniques [41]. However, a controlled randomized protocol would have been necessary to investigate this potential causative relationship.

### Implications for PCHMS Design and Future Research

#### Potential Considerations

This study suggests that there is potential for using the private spaces in a PCHMS (eg, diary and PHR) to enable participants to self-reflect and take action in regard to their health. As participants become more aware of their health status and concerns, they could then utilize the social environment in the PCHMS to (1) seek advice and support from similar others (eg, via online community forum), (2) engage with health professionals (eg, via expert-based question-and-answer forum), (3) verify whether they are “normal” compared to others (eg, via poll), or (4) use health service facilitation tools to connect with formal services for assistance (eg, online appointment booking).

In fact, consumers’ usage patterns of a PCHMS could provide important “signals” of whether help-seeking assistance is needed. Based on the way a consumer uses these social and self-reflective features, the PCHMS could potentially facilitate help-seeking behavior by adaptively managing and promoting the individual’s interactions with other consumers/health care providers, thereby elucidating the path to help-seeking through a method that would be most appropriate at that point in time.

Further, eHealth researchers should consider ways that the “crowd” (ie, social network around a person) can be systematically manipulated so that it can influence health behaviors and outcomes in a positive manner. If a PCHMS were able to “change” the crowd around a person, would that be sufficient for him or her to come into contact with a pivotal person who would encourage help-seeking and early intervention? If so, what “doses” of self-reflection and changes in social network formations are required in an intervention for an individual to take action?

#### Forum

Our social network analysis of online forums has revealed a spectrum of social interaction patterns—from question-and-answer structures to community discussions. It also provides a preliminary examination of how the presence of experts in online forums may change the patterns of communication among consumers.

Current evidence that guides the design of social features in consumer eHealth applications is sparse. More empirical and theoretical studies are needed to investigate ways to design an “optimally social-engineered communication space” [42], according to its intended purpose and the anticipated interaction mode. Choi and colleagues have recently proposed a typology for online Question-Answering (QA) forums with four categories: community-based, collaborative, expert-based QAs, and social [37]. When designing social components of a PCHMS, researchers should consider the following important questions:

(1) Which type of QA online space is most appropriate to address the needs of its audience? What is the optimal mix of participants that would allow interaction/moderation to be sustainable in the long-term?

(2) In a community-based forum, would it be more appropriate to have informed “expert peers” [43] rather than “medical professionals” to act as moderators? What method of moderation is most appropriate in order to encourage participant activity without compromising on the fluidity of interaction, the safety of the space, or the accuracy of the information exchanged?

(3) How can online spaces be optimally designed for different social and communication purposes (eg, with new acquaintances, family and friends, or health professionals)?

#### Poll

The poll was one of the most frequently used features in the PCHMS and was regarded as the most useful and engaging. Yet, its use in consumer eHealth applications is not widespread, its efficacy not thoroughly tested, and it remains unclear how we can effectively design and incorporate social norms information to influence health behaviors. As Christakis and Fowler have demonstrated in the past decade, social networks are associated with health behaviors and outcomes for a variety of conditions (such as happiness, loneliness, depression, and obesity) [44-47]. When applied in the right context, social norms information has shown to significantly influence health behaviors (such as reducing alcohol consumption [48,49]). In addition, Centola has recently demonstrated that homophily (ie, similarity of social contacts) and social network structures can significantly influence *online* health behaviors [50,51].

Yet, to the best of our knowledge, no studies have examined how we can best utilize these social influence findings to inform the design of PCHMSs and other consumer eHealth applications. While previous literature and our own findings suggest that information about social norms (such as via the poll) is associated with significant changes in consumers’ health beliefs and behaviors, eHealth researchers need to examine ways we can effectively utilize social norms information to encourage positive health behaviors. Similarly, there is a need to reduce the risk of “normalizing” negative health behaviors and beliefs, such as in cases when the “norms” may convey an incorrect or misleading view of what is considered healthy.

#### Diary and Personal Health Record

The poll and the forum promote social interactions with other people, whereas the PHR and the diary provide consumers an online private space in which to organize, reflect, and hopefully, advance their health. Usage patterns of the diary suggest that it was primarily used for self-reflection, personal problem solving, and goal setting. On the other hand, the PHR was primarily used for organizational purposes, which included the self-recording of personal health data and past/upcoming tasks.

### Strengths and Limitations

Key strengths of this study include the employment of a multifaceted PCHMS and the utilization of PCHMS usage metrics to identify associations with key consumer behavior outcomes.

This study also presents several limitations: university setting, self-reports, self-entry functionality, causality versus association, and PCHMS engagement measures. First, participants in a university setting may have been more motivated and willing to try new technologies to manage their health than the general population [52,53]. The key limitations are the short duration and high attrition rate. High attrition rates are common in eHealth intervention studies, with a recent systematic review revealing that completion of protocol rates for depression sites ranged from 43% to 99% [54]. One of the possible reasons for the attrition rate of 64.28% (1276/1985) in this study is that participants were asked by email to complete their post-study questionnaire during the long university summer break, when students and staff were not as likely to check their university email. However, the number of participants eligible for analysis is still relatively large (n=709), with 80.7% (572/709) logging into the PCHMS at least once [27], providing a sufficient sample size to analyze whether level of PCHMS usage is associated with consumers’ heath service and help-seeking utilization rates. Overall, future studies conducted in the university setting should strive to commence and complete the study during semester time.

Second, the study relied on self-reports by participants, which have been shown to be acceptable in studies of help-seeking, health service utilization, and mental health related studies among students [55-58]. The PCHMS currently relies on self-entry functionality, which may have caused lower usage of the tool. While it is possible that some participants could have used the PCHMS after visiting the university health services, we validated health service utilization rates by matching self-report from a subset of study participants with their health records at the University Counselling and Psychological Services, where system usage log files indicated that usage of the PCHMS preceded clinic visits.

Third, although findings in this study are limited by its design, the use of a convenience sample, and that we could attribute no causal relationships, our findings concur with Couper and colleagues’ study, which found that website engagement was significantly associated with consumers’ health behaviors [59]. In addition, our previous analyses showed that participants’ pre-study characteristics and well-being ratings were uniformly distributed among different PCHMS log-in frequency thresholds [27], and we have demonstrated in a previous randomized controlled trial that use of the PCHMS is associated with significant uptake of the influenza vaccine [23]. Nevertheless, future studies will need to use a controlled randomized design to allow an interpretation in terms of causality.

Finally, this study focuses on some of the simplest website engagement measures (eg, number of PCHMS log-ins) and differed from previous studies that have used numerous metrics for measuring user engagement, such as number of website visits, time spent on a site, and number of features used [53,60]. Future studies should consider incorporating a qualitative component to elicit participants’ context and reasons (eg, why and how) for engaging with the PCHMS.

### Conclusions

Incorporating the two major trends in consumer eHealth research (ie, PHRs and online social networks) to inform the next generation design of consumer systems requires several novel considerations. This study provides preliminary findings that suggest a PCHMS should include both social and self-reflective features that allow consumers to become familiar with their personal concerns and connect with others to seek help. With the rapid growth of online social networking websites and PHRs, future designs of PCHMSs should explore novel ways in which we can intervene in a person’s level of self-awareness and social network and examine their efficacy as a complex social and self-reflective intervention for health.

## Multimedia Appendix 1

Study protocol and intervention description.

## Multimedia Appendix 2

Personal health record entries entered by participants.

## Abbreviations

COOP/WONCA charts: Charts developed by the Dartmouth Primary Care Cooperative Research Network (COOP) and the World Organization of National Colleges, Academies, and Academic Associations of General Practitioners/Family Physicians (WONCA).
GP: general practitioner
PCHMS: personally controlled health management system
PHR: personal health record
QA: question-answering
