# Supplementary material for: Social and Self-Reflective Use of a Web-Based Personally Controlled Health Management System
Source: J Med Internet Res. 2013 Sep 23;15(9):e211. doi: 10.2196/jmir.2682 (PMC3785989; doi:10.2196/jmir.2682)
Supplement: Supplementary file 1 [file jmir_v15i9e211_app1.pdf]

Appendix 1: Methods

Note: This method section was originally published in:

Lau AYS, Proudfoot J, Andrews A, Liaw ST, Crimmins J, Arguel A, Coiera E
Which Bundles of Features in a Web-Based Personally Controlled Health Management System Are Associated With Consumer Help-Seeking Behaviors for Physical and Emotional Well-Being?
J Med Internet Res 2013;15(5):e79
URL: http://www.jmir.org/2013/5/e79/
doi: 10.2196/jmir.2414
PMID: 23649790

Reproduced under the terms of the Creative Commons Attribution License:
©Annie YS Lau, Judith Proudfoot, Annie Andrews, Siaw-Teng Liaw, Jacinta Crimmins, Amaël Arguel, Enrico Coiera. Originally published in the Journal of Medical Internet Research (http://www.jmir.org), 06.05.2013.

Trial Design and Participants

A one-group pre/posttest online prospective study was conducted over a university academic semester (July to November 2011). Inclusion criteria were (1) aged 18 or above, and (2) with access to the Internet and email at least on a monthly basis.

Study Protocol

Students and staff were approached via email lists and advertisements in online print publications, which described the study and invited interested parties to use a PCHMS called *Healthy.me* developed at the University of New South Wales (UNSW) to manage their physical and emotional well-being for an academic semester. Written informed consent was sought online from each participant. Participants then completed a 15-minute online pre-study survey, followed by a 5-minute mandatory online tutorial about *Healthy.me* prior to using the site. At study completion (end of semester), participants received an email asking them to complete a 15-minute online post-study survey. Two follow-up emails 5 days apart were sent as reminders to noncompleters. Those who completed all surveys were entered into a draw for an AU$500 gift voucher. A researcher was available via a dedicated telephone line and email to answer participants' questions and concerns during the study. Ethics approval was obtained from the UNSW ethics committee.

Measures

At baseline, demographic information (such as age and gender) was collected, as well as information about their use of social networking websites, use of the Internet to find health-related information, and visits to a health professional (including whether they visited prior to the study a health care professional, University Health Service, and the University Counselling and Psychological Services).
In the pre- and post-study questionnaires, measures 1-3 were administered and additional measures (4-5) were administered in the postintervention questionnaire:

1. *COOP/WONCA charts* were used to evaluate participants' functional status, defined as physical, emotional, and social status. These scales, which have been demonstrated

to be a valid and feasible one-time screening assessment for mental disorders in primary care [34], measure six domains, namely physical fitness, feelings, daily activities, social activities, change in health, and overall health. Responses are via a 1-5 Likert-scale where higher scores indicate a poorer functional status.

2. *Well-being self-ratings and lifestyle intention*: adapted from the last question in the standardized instrument EUROQOL (EQ-5D) [35], which measures health status, participants were asked to rate their physical and emotional well-being on a scale from 0 to 100. They were also asked to select one of four statements that best describes their intention to practice a lifestyle that benefits their well-being according to the transtheoretical model of behavior change [17].
3. *Health advice-seeking and health advice-providing networks*: adapted from the Norbeck Social Support Questionnaire [36], participants were asked to nominate up to 5 people they have sought advice from, or provided advice to, before and during the study.
4. *Help-seeking behaviors and health service utilization*: Help-seeking is defined as the behavior of actively seeking assistance [37], regardless of whether the source is informal or formal. A new scale was developed by the authors, adapted from the Actual Help-seeking Questionnaire (AHSQ) [37]. The scale covers help-seeking behaviors for physical and emotional well-being, informal and formal sources, as well as for self or others.
5. *Feedback on Healthy.me*: participants were asked to provide feedback on their overall experience of using *Healthy.me*, as well as their feedback on specific features on the website, using a range of scale items such as Likert scale, free-text comments, and checkbox answer options.

PCHMS Usage Metrics

A recent review by Danaher and Seeley [38] concluded there is no single, universally accepted measure for website usage, and researchers are still debating the best methods for defining and measuring website engagement [38].

In this study, we used simple website engagement measures to track participants' activity on the website (ie, PCHMS login frequency and whether participants accessed, or did not access, each website feature). These measures were used to assess whether (1) there was a dose-response effect, that is, was the frequency of PCHMS login associated with rates of health service utilization and help-seeking behaviors, and whether (2) access to PCHMS feature(s) (ie, journey, personal health record, forum, poll, diary, and/or online appointment service) was associated with participants' health service utilization and help-seeking behaviors for physical and/or emotional well-being.

PCHMS Web logs were analyzed to determine whether participants accessed (or did not access) any of the features at any time during the study. Some of these website engagement measures have previously been used to measure user engagement of PHR systems [39].

Intervention

Healthy.me

Healthy.me was iteratively developed, and its first version was tested in other settings such as in vitro fertilization and influenza vaccination [42,43]. The first version contained features such as journey, the personal health record, and online appointment booking with the

university primary care service. The version of *Healthy.me* (version 2.0) that was used in this study contained the above-mentioned features as well as online appointment booking with the university primary care and counselling services, a diary, forum, and poll. Details of each feature are described below:

1. Personal Health Record (PHR) for self-recording of medical test results, medications, scheduled appointments, and personnel looking after one's health (see Figure 1).
2. Online appointment booking with the University Health Service (primary care) and the UNSW Counselling and Psychological Services (sent via email using the "Book now" button in the PCHMS).
3. Diary for participants to write down their thoughts about their health. By default, the diary is private. However, participants can select to share their diary with all participants enrolled in the PCHMS.
4. Social communication spaces, which support interaction across the continuum of care between fellow participants and clinicians. Features include the poll system and forums moderated by clinicians. Poll system in which participants answer simple health questions (eg, how much sleep did you get last night?), where they can view and compare their response with other participants' aggregated answers in graph format (Figure 2). Forums moderated by clinicians (a primary care physician and a psychologist), where participants can either post their entries on the forum or send one-on-one email messages to other participants in the PCHMS (including clinicians). Guidelines on forum use and the protocol for responding to concerns reported in the forum were approved from the UNSW ethics committee. Posts sent by participants to the "Report concern" feature on the forum were emailed to clinical and research personnel during the study, who investigated any reported concerns. A Uniform Resource Locator (URL) available in the email to the dedicated staff allowed them to withdraw the forum post. The primary care physician and the psychologist not only moderated the forum but were also available to answer questions posted on the forums. No harm from the use of the forum or the PCHMS was reported by participants during the study.
5. Journeys that provide information for consumer participants to engage with clinicians and health services in an actionable way. Participants in this study had access to four well-being journeys for physical and emotional well-being: "Stay Healthy", "Stressed out?", "Feeling Anxious about the Exams?", and "My Emotional Well-being Program".

The four well-being journeys for physical and emotional well-being were designed and developed in consultation with University Counselling and Psychological Services psychologists and University Health Service primary care physicians, utilizing evidence-based consumer education material routinely used at UNSW to promote physical and emotional well-being. Written in youth-friendly language, using evidence-based mental health, psychoeducational, and psychosocial material, the journeys consisted of skills-focused content delivered online, as well as well-being workshops that participants could attend in-person at the University Counselling and Psychological Services. Participants could learn about mindfulness meditation, anxiety management, time management, and stress management at these workshops.

Journeys were delivered via the PCHMS at four pivotal time-points during a university academic semester (ie, beginning of semester, 4 weeks into semester, after mid-semester break, and before exams) to address physical and emotional well-being concerns likely to be concerning participants at each time-point. Participants were alerted with an email when a

new journey became available on the PCHMS. These journeys provided task specific knowledge in an actionable way. For example, as participants read the journey for advice on physical or emotional well-being, they could immediately:

- book an appointment with a university primary care physician or a psychologist from the journey page,
- register to attend a well-being workshop,
- post a question on a forum to seek advice from fellow participants or a clinician (primary care physician or a psychologist), or
- send themselves an email reminder to do so later.

A pilot study was conducted in a controlled setting with 15 university staff and students of different ages, gender, and familiarity with computers to test the intervention, the measures, and the research design. Substantive usability issues were resolved before recruiting participants in their real-life setting.
