# Supplementary material for: Social and Self-Reflective Use of a Web-Based Personally Controlled Health Management System
Source: J Med Internet Res. 2013 Sep 23;15(9):e211. doi: 10.2196/jmir.2682 (PMC3785989; doi:10.2196/jmir.2682)
Supplement: Supplementary file 2 [file jmir_v15i9e211_app2.pdf]

**Appendix 2**

*Note: Personal identifiable information have been removed or replaced*

**Appendix Table 1**. Examples of medication entries entered by participants in their personal health records

| description | route | dose | frequency | start_time | stop_time | Remarks |
|---|---|---|---|---|---|---|
| Thompson's Cholesterol Manager | Orally | 2 tablets before breakfast and 2 before dinner after 1 week drops down to 1 before breakfast and dinner. | Daily | 17/07/2010 00:00 | (null) | 2 tablets prior to breakfast and dinner for first week and then after 1 week 1 tablet prior to breakfast and dinner. |
| Pulmicort Turbuhaler 100 microgram/actuation inhalation: powder for 200 actuations | Turbuhaler - inhale | 1 click | Once a week (Sundays) | (null) | (null) | For my asthma.. but only need this in winter and spring when my asthma is at it's worst or most susceptible I guess to being triggered. |

| | | | | | | |
|---|---|---|---|---|---|---|
| Zoloft 100 mg tablet: film-coated 30 tablets | orally | 2 | Daily | 1/11/2009 00:00 | (null) | severe depression PTSD anxiety |
| Minomycin 50 mg tablet: film-coated 28 tablets | Oral | 1 tablet | once | 1/06/2011 00:00 | 9/08/2011 00:00 | I have been taking it on and off for the past 2-3 years to control my acne and it does control it but doesn't stop it. |
| Yasmine | Swallow tablet with water | one tablet | Once a day | (null) | (null) | |

**Appendix Table 2**. Examples of healthcare team member entries entered by participants in their personal health records

| name | role | Location | contact_details | comment |
|---|---|---|---|---|
| Dr <firstname surname> | Dentist | <Full address> | <Full telephone number> | 6 monthly clean and check up |
| Dr <firstname surname> | Consultant GASTROENTEROLOGIST | <Full address> | <Full telephone number> | Gastroscopy March 2012. |
| Dr <firstname | Cardiologist | <Full address> | <Full telephone | Complete check November 2011 |

| | | | | |
|---|---|---|---|---|
| surname> | | | number> | with all results OK. Including exercise stress test, ECG, test for cardiomyopathy |
| <firstname surname> | Boyfriend | Home | <Full telephone number> | |
| <firstname surname> | sports and exercise medicine physiican | <Full address> | <Full telephone number> | wwww.orthospor ts |
| DR <firstname surname> | GP | doctors on <street suburb> | <Full telephone number> | better than dr <surname> |
| Mr <firstname surname> | Chiropractor | Mosman | | Current treatment for chronic neck pain |
| Mr <surname> | Osteopath | Mosman | | Reduced symptoms but did not help long term pain |
| Ms <surname> | Physiotherapist | Mosman | | Helped to reduce pain during the HSC but did not help in long term |
| Sydney Breast Clinic | scan and bone densitometry | <Full address> | <Full telephone number> | |

**Appendix Table 3**. Examples of pathology test result entries entered by participants in their personal health records

| test_descript | date_time | doctor | provider | result | comment |
|---|---|---|---|---|---|

| | | | | | |
|---|---|---|---|---|---|
| **ion** | | | | | |
| Blood Test | 4/01/2010 | Dr <surname> | Douglass Hanly Moir | (null) | High results show infection present but not glandular fever |
| Serum HBV Ab screening | 12/07/2011 | Dr <firstname surname> | Laverty | Neg | Not immune to HBV |
| Blood Test | 27/07/2011 | Ms <surname> | Mosman Clinic | low in iron high in vitamin D CRP (high sens) | Need to increase intake of red meat |
| FBC + IRON + THYROID + VITAMINS | 1/08/2011 | Dr <firstname> | | vitamin D deficient otherwise normal | |
| Ferritin | 17/05/2011 | <surname> | Hanly-moir | 11 | |
| Urine Test (Dipstick For Protein) | 17/08/2011 | Dr <surname> | | Dehydrated light blood | |
| Blood test (Full) | 1/07/2011 | <firstname surname> | Laverty Pathology | | All fine but one cholesterol reading slightly high. Doctor said it's no concern right now. |
| PAP smear | 1/08/2011 | dr <surname> | | CLEAR | |

**Appendix Table 4**. Examples of procedure entries entered by participants in their personal health records

| **procedure** | **clinician** | **location** | **comment** | **provider** | **result** |
|---|---|---|---|---|---|
| Mole removal | Dr <firstname surname> | | all good | | |
| gall bladder removal | DR <firstname surname> | | all removed ok some diet questions | Dr <firstname surname> | |
| Fractured ankle | Dr <firstname surname> | | Ffracture in Left ankle no surgery required healed OK but Feel uneasy or unsteady | RPA Fracture Clinic | |
| X-ray | Dentist | | X-ray for wisdom teeth | | |
| Vision test | | | Power increased in right eye by 0.5 | Specsavers - Canberra city | RE -3 -0.25 85<br>LE -2.5 -0.25 20 |
| LETZ | Dr <surname> | | | Kogorah Public Hospital | |
| cryotherapy | <firstname surname> | | Cryotherapy done next on 9th sept | <firstname surname> | |
| Right Knee Arthroscopy | Dr <firstname surname> | | | Dr <firstname surname> | Repair of medial cartledge (20% removed) |

| | | | | | |
|---|---|---|---|---|---|
| | | | | | ACL noted as in tact |
| Mammogram | | | | 73 | |
| Colonoscopy | | | | 74 | |

**Appendix Table 5**. Examples of imaging test entries entered by participants in their personal health records

| **Imaging test** | **date_time** | **doctor** | **provider** | **result** | **comment** |
|---|---|---|---|---|---|
| mammogram | 16/02/2009 | Dr <firstname surname> Bondi Junction | Breast screen NSW | NAD | |
| MRI - June 11 Right Knee | 7/06/2011 | Dr <firstname surname> | St George MRI | Medial meniscus tear insufficient blow supply to ACL (weakened) | |
| Pelvic ultrasound | 5/06/2011 | | X-Ray Bondi Jct | | everything seemed to be ok |
| DeNTAL xRAY | 3/08/2011 | <firstname surname> | | | |
| X-ray | 11/07/2011 | Mr <surname> | Sydney Radiology | Can be seen from x-rays a poor neck structure | Due to chronic neck pain |
| X-ray | 28/07/2011 | | | not broken or displaced | for Coccyx |
| XRAY LUNG | 2/02/2011 | Dr <firstname | | | CLEAR |

| | | | | | |
|---|---|---|---|---|---|
| | | surname> | | | |
